# *catena*-Poly[[diaqua­nickel(II)]-bis­(μ-pyridine-4-sulfinato)-κ^2^ *N*,*O*;κ^2^ *O*,*N*]

**DOI:** 10.1107/S1600536809024258

**Published:** 2009-07-01

**Authors:** Zhong-Xiang Du, Su-Ning Ji

**Affiliations:** a Department of Chemistry, Luoyang Normal University, Luoyang, Henan 471022, People’s Republic of China

## Abstract

In the title coordination polymer, [Ni(C_5_H_4_NO_2_S)_2_(H_2_O)_2_]_*n*_, the Ni^II^ ion is located on an inversion centre and is octa­hedrally coordinated by two N and two O atoms of four symmetry-related and deprotonated pyridine-4-sulfinate (ps) ligands together with two water mol­ecules in axial positions. The ps^−^ anions, acting as μ_2_-bridging ligands, link neighbouring Ni^II^ ions into a chain structure along the *c* axis. These polymeric chains are extended into a three-dimensional framework *via* inter­molecular O—H⋯O hydrogen bonds with participation of the water mol­ecules.

## Related literature

For metal complexes derived from pyridine-4-sulfonic acid, see: Lü *et al.* (2007 ▶); Leslie & George (2005*a* ▶,*b* ▶).
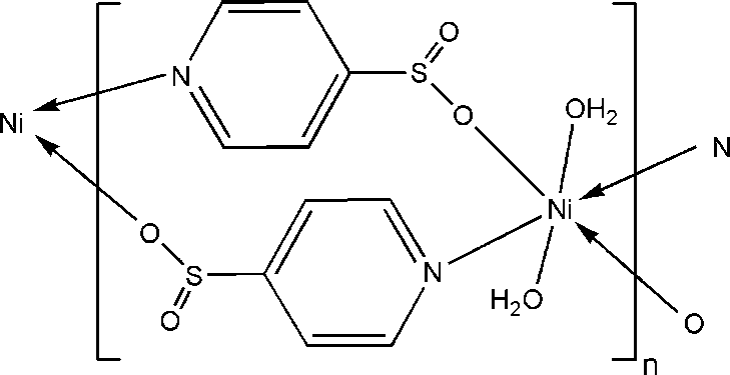


## Experimental

### 

#### Crystal data

[Ni(C_5_H_4_NO_2_S)_2_(H_2_O)_2_]
*M* *_r_* = 379.05
Triclinic, 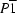

*a* = 6.403 (5) Å
*b* = 7.309 (5) Å
*c* = 7.602 (5) Å
α = 96.784 (8)°
β = 95.140 (8)°
γ = 107.709 (8)°
*V* = 333.6 (4) Å^3^
*Z* = 1
Mo *K*α radiation
μ = 1.80 mm^−1^
*T* = 296 K
0.25 × 0.17 × 0.14 mm

#### Data collection

Bruker APEXII CCD area-detector diffractometer
Absorption correction: multi-scan (*SADABS*; Sheldrick, 1996 ▶) *T*_min_ = 0.662, *T*_max_ = 0.787
2417 measured reflections
1180 independent reflections
1043 reflections with *I* > 2σ(*I*)
*R*_int_ = 0.017

#### Refinement

*R*[*F* ^2^ > 2σ(*F* ^2^)] = 0.031
*wR*(*F* ^2^) = 0.080
*S* = 1.00
1180 reflections
97 parameters
H-atom parameters constrained
Δρ_max_ = 0.68 e Å^−3^
Δρ_min_ = −0.36 e Å^−3^

### 

Data collection: *APEX2* (Bruker, 2004 ▶); cell refinement: *SAINT* (Bruker, 2004 ▶); data reduction: *SAINT*; program(s) used to solve structure: *SHELXS97* (Sheldrick, 2008 ▶); program(s) used to refine structure: *SHELXL97* (Sheldrick, 2008 ▶); molecular graphics: *SHELXTL* (Sheldrick, 2008 ▶); software used to prepare material for publication: *SHELXTL*.

## Supplementary Material

Crystal structure: contains datablocks global, I. DOI: 10.1107/S1600536809024258/at2818sup1.cif

Structure factors: contains datablocks I. DOI: 10.1107/S1600536809024258/at2818Isup2.hkl

Additional supplementary materials:  crystallographic information; 3D view; checkCIF report

## Figures and Tables

**Table 1:** Selected geometric parameters (Å, °)

Ni1—N1^i^	2.008 (2)
Ni1—O3	2.026 (2)
Ni1—O1	2.362 (2)

Symmetry code: (i) 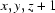.

**Table 2:** Hydrogen-bond geometry (Å, °)

*D*—H⋯*A*	*D*—H	H⋯*A*	*D*⋯*A*	*D*—H⋯*A*
O1—H1*W*⋯O2^iv^	0.84	2.00	2.826 (3)	168
O1—H2*W*⋯O2^v^	0.84	2.00	2.827 (3)	169

Symmetry codes: (iv) 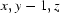; (v) 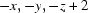.

**Fig. 1.**
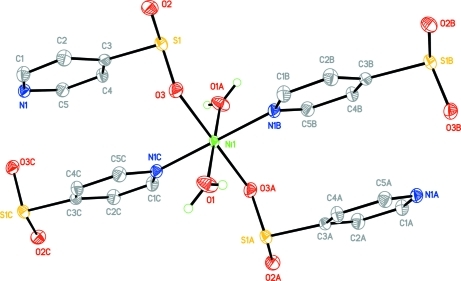
The coordination environment of NiII ion in (I).Displacement ellipsoids are drawn at the 30% probability level. Symmetry codes: (A) (1 - x, -y, 2 - z);(B) (x, y, 1 + z); (C) (1 - x, -y, 1 - z).

**Fig. 2.**
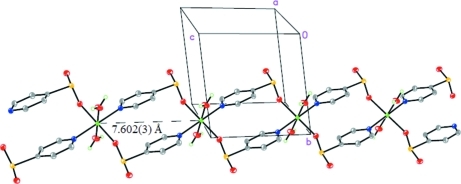
The chain structure of (I) along c axis. H atoms on C atoms have been omitted.

**Fig. 3.**
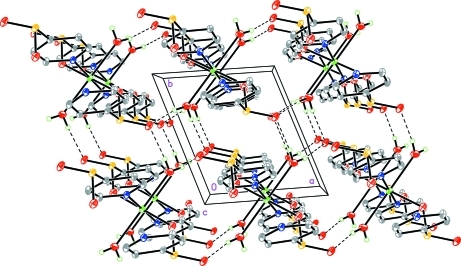
Packing diagram for (1), showing hydrogen bonds as dashed lines in ab plane. H atoms on C have been deleted.

## supplementary crystallographic information

### Comment

It is well known that sulfinic acid is not stable compared with sulfonic acid, so it is much difficult to obtain complexes of sulfinic acid as they are easy to be oxidized. In the previous literatures, several metal complexes derived from pyridine-4-sulfonic acid have been reported (Leslie & George, 2005a,b; Lü *et al.*,  2007), whereas the complexes of pyridine-4-sulfinic acid has been not seen so far. Here we describe a nickel(II) complex from pyridine-4-sulfinic acid, (I), (Fig. 1).

The Ni^II^ ion locates on a centre of symmetry and is in a distorted octahedral geometry with two water ligands in axial *trans* positions and two N and two O atoms of four symmetry-related ps^-^ ligands in equatorial plane (Table 1). Each ps^-^ ligand connects two Ni^II^ ions and thus forms one-dimensional chain structure along *c* axis (Fig.2), with adjacent Ni···Ni separation distance of 7.602 (3) Å.

Water molecules take part in hydrogen bonds as double donor, and S═O of ps^-^ ligands acts only as a single acceptor (Table 2, Fig.3). Hydrogen bonds interactions stabilizes and extends chain structure of (I) into a three-dimensional network.

### Experimental

A solution of NiCl_2_. 6H_2_O (1 mmol, 0.238 g) in anhydrous ethanol (10 ml) was injected dropwise into a solution of Hps (2 mol, 0.286 g) in methanol (15 ml) under argon. The resulting mixture was stirred at 343 K for 4 h, then cooled to room temperature. After filtration, the filtrate was left to stand at room temperature for slow evaporation. Green block-shaped crystals suitable for X-ray diffraction were obtained in a yield of 17%. Analysis, found: C 31.58, H 3.11, N 7.45, S 16.93%; C_10_H_12_N_2_NiO_6_S_2_ requires: C 31.66, H 3.17, N 7.39, S 16.88%.

### Refinement

H atoms bonded to C were positioned geometrically with C—H distance of 0.93 Å, and treated as riding atoms, with *U*_iso_(H)=1.2*U*_eq_(C). The O—H hydrogen atom was located in a difference Fourier map and refined isotropically.

### Crystal data

**Table:**

[Ni(C_5_H_4_NO_2_S)_2_(H_2_O)_2_]	*Z* = 1
*M**_r_* = 379.05	*F*(000) = 194
Triclinic, *P*1	*D*_x_ = 1.887 Mg m^−^^3^
Hall symbol: -P 1	Mo *K*α radiation, λ = 0.71073 Å
*a* = 6.403 (5) Å	Cell parameters from 1414 reflections
*b* = 7.309 (5) Å	θ = 2.7–27.9°
*c* = 7.602 (5) Å	µ = 1.80 mm^−^^1^
α = 96.784 (8)°	*T* = 296 K
β = 95.140 (8)°	Block, green
γ = 107.709 (8)°	0.25 × 0.17 × 0.14 mm
*V* = 333.6 (4) Å^3^	

### Data collection

**Table:**

Bruker APEXII CCD area-detector diffractometer	1180 independent reflections
Radiation source: fine-focus sealed tube	1043 reflections with *I* > 2σ(*I*)
graphite	*R*_int_ = 0.017
φ and ω scans	θ_max_ = 25.5°, θ_min_ = 3.0°
Absorption correction: multi-scan (*SADABS*; Sheldrick, 1996)	*h* = −7→7
*T*_min_ = 0.662, *T*_max_ = 0.787	*k* = −8→8
2417 measured reflections	*l* = −9→9

### Refinement

**Table:**

Refinement on *F*^2^	Primary atom site location: structure-invariant direct methods
Least-squares matrix: full	Secondary atom site location: difference Fourier map
*R*[*F*^2^ > 2σ(*F*^2^)] = 0.031	Hydrogen site location: inferred from neighbouring sites
*wR*(*F*^2^) = 0.080	H-atom parameters constrained
*S* = 1.00	*w* = 1/[σ^2^(*F*_o_^2^) + (0.0581*P*)^2^] where *P* = (*F*_o_^2^ + 2*F*_c_^2^)/3
1180 reflections	(Δ/σ)_max_ < 0.001
97 parameters	Δρ_max_ = 0.68 e Å^−^^3^
0 restraints	Δρ_min_ = −0.36 e Å^−^^3^

### Special details

**Table:**

Geometry. All e.s.d.'s (except the e.s.d. in the dihedral angle between two l.s. planes)are estimated using the full covariance matrix. The cell e.s.d.'s are takeninto account individually in the estimation of e.s.d.'s in distances, anglesand torsion angles; correlations between e.s.d.'s in cell parameters are onlyused when they are defined by crystal symmetry. An approximate (isotropic)treatment of cell e.s.d.'s is used for estimating e.s.d.'s involving l.s. planes.
Refinement. Refinement of *F*^2^ against ALL reflections. The weighted *R*-factor *wR* and goodness of fit *S* are based on *F*^2^, conventional *R*-factors *R* are based on *F*, with *F* set to zero for negative *F*^2^. The threshold expression of *F*^2^ > σ(*F*^2^) is used only for calculating *R*-factors(gt) *etc*. and is not relevant to the choice of reflections for refinement. *R*-factors based on *F*^2^ are statistically about twice as large as those based on *F*, and *R*- factors based on ALL data will be even larger.

### Fractional atomic coordinates and isotropic or equivalent isotropic displacement parameters (Å^2^)

**Table:**

	*x*	*y*	*z*	*U*_iso_*/*U*_eq_	
Ni1	0.5000	0.0000	1.0000	0.02196 (18)	
S1	0.37032 (11)	0.32818 (9)	0.81172 (8)	0.0282 (2)	
O1	0.1697 (3)	−0.2599 (3)	0.9900 (3)	0.0432 (5)	
H1W	0.1594	−0.3595	0.9190	0.065*	
H2W	0.0660	−0.2844	1.0522	0.065*	
O2	0.1653 (3)	0.3862 (3)	0.7920 (3)	0.0374 (5)	
O3	0.3059 (3)	0.1287 (3)	0.8709 (2)	0.0330 (4)	
N1	0.4591 (3)	0.1289 (3)	0.2369 (3)	0.0257 (5)	
C1	0.2579 (4)	0.1289 (4)	0.2770 (4)	0.0306 (6)	
H1	0.1377	0.0841	0.1873	0.037*	
C2	0.2257 (5)	0.1933 (4)	0.4472 (4)	0.0320 (6)	
H2	0.0857	0.1920	0.4721	0.038*	
C3	0.4053 (4)	0.2601 (3)	0.5805 (3)	0.0250 (5)	
C4	0.6131 (4)	0.2690 (4)	0.5387 (3)	0.0271 (6)	
H4	0.7370	0.3191	0.6247	0.033*	
C5	0.6316 (4)	0.2014 (4)	0.3656 (4)	0.0304 (6)	
H5	0.7713	0.2066	0.3369	0.036*	

### Atomic displacement parameters (Å^2^)

**Table:**

	*U*^11^	*U*^22^	*U*^33^	*U*^12^	*U*^13^	*U*^23^
Ni1	0.0291 (3)	0.0292 (3)	0.0131 (2)	0.0168 (2)	0.00585 (17)	0.00249 (17)
S1	0.0353 (4)	0.0309 (4)	0.0215 (4)	0.0151 (3)	0.0082 (3)	0.0004 (3)
O1	0.0457 (12)	0.0320 (11)	0.0528 (13)	0.0108 (9)	0.0236 (10)	0.0015 (9)
O2	0.0473 (12)	0.0441 (11)	0.0339 (11)	0.0299 (10)	0.0164 (9)	0.0075 (9)
O3	0.0414 (11)	0.0401 (10)	0.0251 (9)	0.0212 (9)	0.0098 (8)	0.0081 (8)
N1	0.0288 (11)	0.0309 (11)	0.0210 (11)	0.0141 (9)	0.0055 (8)	0.0047 (9)
C1	0.0284 (13)	0.0412 (15)	0.0254 (14)	0.0162 (11)	0.0033 (11)	0.0040 (11)
C2	0.0277 (13)	0.0435 (15)	0.0299 (15)	0.0176 (12)	0.0089 (11)	0.0047 (12)
C3	0.0328 (13)	0.0246 (12)	0.0217 (13)	0.0134 (10)	0.0079 (10)	0.0047 (10)
C4	0.0271 (13)	0.0327 (13)	0.0217 (13)	0.0106 (11)	0.0036 (10)	0.0015 (10)
C5	0.0304 (14)	0.0377 (15)	0.0275 (15)	0.0160 (12)	0.0084 (11)	0.0053 (11)

### Geometric parameters (Å, °)

**Table:**

Ni1—N1^i^	2.008 (2)	N1—C5	1.336 (4)
Ni1—N1^ii^	2.008 (2)	N1—C1	1.350 (3)
Ni1—O3^iii^	2.026 (2)	N1—Ni1^iv^	2.008 (2)
Ni1—O3	2.026 (2)	C1—C2	1.379 (4)
Ni1—O1^iii^	2.362 (2)	C1—H1	0.9300
Ni1—O1	2.362 (2)	C2—C3	1.386 (4)
S1—O2	1.498 (2)	C2—H2	0.9300
S1—O3	1.523 (2)	C3—C4	1.380 (4)
S1—C3	1.821 (3)	C4—C5	1.378 (4)
O1—H1W	0.8350	C4—H4	0.9300
O1—H2W	0.8371	C5—H5	0.9300
			
N1^i^—Ni1—N1^ii^	180.000 (1)	H1W—O1—H2W	109.3
N1^i^—Ni1—O3^iii^	90.55 (9)	S1—O3—Ni1	128.93 (12)
N1^ii^—Ni1—O3^iii^	89.45 (9)	C5—N1—C1	118.2 (2)
N1^i^—Ni1—O3	89.45 (9)	C5—N1—Ni1^iv^	119.50 (18)
N1^ii^—Ni1—O3	90.55 (9)	C1—N1—Ni1^iv^	121.99 (18)
O3^iii^—Ni1—O3	180.000 (1)	N1—C1—C2	121.9 (2)
N1^i^—Ni1—O1^iii^	92.01 (8)	N1—C1—H1	119.1
N1^ii^—Ni1—O1^iii^	87.99 (8)	C2—C1—H1	119.1
O3^iii^—Ni1—O1^iii^	85.24 (9)	C1—C2—C3	118.9 (2)
O3—Ni1—O1^iii^	94.76 (9)	C1—C2—H2	120.5
N1^i^—Ni1—O1	87.99 (8)	C3—C2—H2	120.5
N1^ii^—Ni1—O1	92.01 (8)	C4—C3—C2	119.5 (2)
O3^iii^—Ni1—O1	94.76 (9)	C4—C3—S1	119.35 (19)
O3—Ni1—O1	85.24 (9)	C2—C3—S1	121.1 (2)
O1^iii^—Ni1—O1	180.0	C3—C4—C5	118.0 (2)
O2—S1—O3	107.19 (12)	C3—C4—H4	121.0
O2—S1—C3	102.55 (12)	C5—C4—H4	121.0
O3—S1—C3	99.77 (11)	N1—C5—C4	123.3 (2)
Ni1—O1—H1W	114.8	N1—C5—H5	118.3
Ni1—O1—H2W	135.0	C4—C5—H5	118.3
			
O2—S1—O3—Ni1	−157.49 (13)	C1—C2—C3—S1	−174.8 (2)
C3—S1—O3—Ni1	96.00 (15)	O2—S1—C3—C4	155.44 (19)
N1^i^—Ni1—O3—S1	−93.25 (15)	O3—S1—C3—C4	−94.3 (2)
N1^ii^—Ni1—O3—S1	86.75 (15)	O2—S1—C3—C2	−26.8 (2)
O1^iii^—Ni1—O3—S1	−1.28 (15)	O3—S1—C3—C2	83.4 (2)
O1—Ni1—O3—S1	178.72 (15)	C2—C3—C4—C5	−3.1 (4)
C5—N1—C1—C2	−2.8 (4)	S1—C3—C4—C5	174.71 (19)
Ni1^iv^—N1—C1—C2	170.9 (2)	C1—N1—C5—C4	2.7 (4)
N1—C1—C2—C3	0.1 (4)	Ni1^iv^—N1—C5—C4	−171.20 (19)
C1—C2—C3—C4	2.9 (4)	C3—C4—C5—N1	0.3 (4)

Symmetry codes: (i) −*x*+1, −*y*, −*z*+1; (ii) *x*, *y*, *z*+1; (iii) −*x*+1, −*y*, −*z*+2; (iv) *x*, *y*, *z*−1.

### Hydrogen-bond geometry (Å, °)

**Table:**

*D*—H···*A*	*D*—H	H···*A*	*D*···*A*	*D*—H···*A*
O1—H1W···O2^v^	0.84	2.00	2.826 (3)	168
O1—H2W···O2^vi^	0.84	2.00	2.827 (3)	169

Symmetry codes: (v) *x*, *y*−1, *z*; (vi) −*x*, −*y*, −*z*+2.
